# Molecular Strategy to Reduce *In Vivo* Collagen Barrier Promotes Entry of NCX1 Positive Inducible Pluripotent Stem Cells (iPSC^NCX1+^) into Ischemic (or Injured) Myocardium

**DOI:** 10.1371/journal.pone.0070023

**Published:** 2013-08-21

**Authors:** Wei Huang, Bo Dai, Zhili Wen, Ronald W. Millard, Xi-Yong Yu, Kristin Luther, Meifeng Xu, Ting C. Zhao, Huang-Tian Yang, Zhihua Qi, Kathleen LaSance, Muhammad Ashraf, Yigang Wang

**Affiliations:** 1 Department of Pathology and Laboratory Medicine, University of Cincinnati Medical Center, Cincinnati, Ohio, United States of America; 2 Infectious Disease Hospital of Nanchang University, Nanchang, Jiangxi, China; 3 Department of Pharmacology and Cell Biophysics, College of Medicine, University of Cincinnati Medical Center, Cincinnati, Ohio, United States of America; 4 Medical Research Center of Guangdong General Hospital, Guangdong Academy of Medical Sciences, Guangzhou, Guangdong, China; 5 Cardiovascular Laboratories, Department of Surgery, Boston University Medical School, Roger William Medical Center, Providence, Rhode Island, United States of America; 6 Laboratory of Stem Cell Biology, Institute of Health Sciences, Shanghai Institutes for Biological Sciences, Chinese Academy of Sciences and Shanghai Jiao Tong University School of Medicine, Shanghai, China; 7 Department of Radiology, University of Cincinnati, Cincinnati, Ohio, United States of America; Northwestern University, United States of America

## Abstract

**Objective:**

The purpose of this study was to assess the effect of collagen composition on engraftment of progenitor cells within infarcted myocardium.

**Background:**

We previously reported that intramyocardial penetration of stem/progenitor cells in epicardial patches was enhanced when collagen was reduced in hearts overexpressing adenylyl cyclase-6 (AC6). In this study we hypothesized an alternative strategy wherein overexpression of microRNA-29b (miR-29b), inhibiting mRNAs that encode cardiac fibroblast proteins involved in fibrosis, would similarly facilitate progenitor cell migration into infarcted rat myocardium.

**Methods:**

*In vitro*: A tri-cell patch (Tri-P) consisting of cardiac sodium-calcium exchanger-1 (NCX1) positive iPSC (iPSC^NCX1+^), endothelial cells (EC), and mouse embryonic fibroblasts (MEF) was created, co-cultured, and seeded on isolated peritoneum. The expression of fibrosis-related genes was analyzed in cardiac fibroblasts (CFb) by qPCR and Western blot. *In vivo*: Nude rat hearts were administered mimic miRNA-29b (miR-29b), miRNA-29b inhibitor (Anti-29b), or negative mimic (Ctrl) before creation of an ischemically induced regional myocardial infarction (MI). The Tri-P was placed over the infarcted region 7 days later. Angiomyogenesis was analyzed by micro-CT imaging and immunofluorescent staining. Echocardiography was performed weekly.

**Results:**

The number of green fluorescent protein positive (GFP^+^) cells, capillary density, and heart function were significantly increased in hearts overexpressing miR-29b as compared with Ctrl and Anti-29b groups. Conversely, down-regulation of miR-29b with anti-29b *in vitro* and *in vivo* induced interstitial fibrosis and cardiac remodeling.

**Conclusion:**

Overexpression of miR-29b significantly reduced scar formation after MI and facilitated iPSC^NCX1+^ penetration from the cell patch into the infarcted area, resulting in restoration of heart function after MI.

## Introduction

In response to MI, cardiac remodeling is activated by structural rearrangement and involves cardiomyocyte hypertrophy, cell death, myofibroblast differentiation, collagen types I and III deposition, fibrosis, and scar formation. Fibrosis and activation of cardiac fibroblasts, the major non-myocyte component (90%) of heart tissue [1], are primary events in ventricular remodeling, rather than a secondary response to cardiac myocyte injury [2]. Fibroblast activation is defined as a transition process wherein fibroblasts lose their characteristics and acquire a myofibroblast phenotype. Specifically, the spindle-like myofibroblasts exhibit a high concentration of smooth muscle α-actin (SMA), and enhanced secretion of ECM proteins, such as collagen I and III, which contribute to fibrotic scar formation. Although cardiac fibrosis is essential for tissue healing after MI, the advanced interstitial fibrosis in the infarcted area and in border zone of the heart reduces the basal and dynamic range of cardiac contractility, diminishes coronary flow reserve, and increases the risk of arrhythmia [3]. Therefore, functional benefits of anti-fibrotic therapy on post-MI heart failure are recognized [4]. For MI and subsequent heart failure, the benefits of conventional treatment are limited due to the inability of the myocardium to regenerate. However, with advancements in the field of regenerative medicine, the potential for stem/progenitor cell-based reconstruction after MI to restore function to failing hearts has been demonstrated.

The two most common routes of cell delivery for myocardial therapy are intravenous and direct intramyocardial injection into an infarcted area. However, it is difficult to control the deposition of grafted cells using these methods. In addition, assuring that transplanted progenitor cells can penetrate and survive in the ischemic myocardial microenvironment is challenging because of a severely compromised nutrient supply, as well as low oxygen and pH levels. Delivery of cells in tissue-like structures that preserve cellular attachments can increase cell delivery efficiency and reduce cell death [5], [6]. With this in mind, we applied a Tri-P, containing CM (to restore heart contractility), EC (to build new blood vessels) and MEF (to provide structural support and cytokines) to the epicardial surface of the infarcted myocardium.

Scar formation at the site of the infarction and interstitial fibrosis of adjacent myocardium serves as a barrier to penetration, engraftment, and survival of progenitor cells from the Tri-cell patch, preventing myocardial repair and leading to the loss of contractile function. For this reason, feasible strategies to directly decrease collagen deposition in the infarct tissue might benefit progenitor cell-based therapy and offer the greatest potential benefit for heart function improvement following MI [6], [7].

Cardiac fibrosis is thought to be mediated by a combination of mechanical and cytokine factors that act on cardiac fibroblasts. Recent studies have demonstrated that several miRNAs play an important role in the regulation of cardiac fibrosis [3], [8]. MicroRNAs (miRNAs) comprise a broad class of small non-coding RNAs that have been demonstrated to modulate cellular processes in a variety of developmental and physiological processes by controlling the expression of mRNA targets [9], [10]. Among myocardial infarction-regulated miRNA members, the miR-29 family (miR-29a, miR-29b copy 1 and copy 2, and miR-29c) is down-regulated in the peri-infarct region of the heart [8], which is associated with collagen production by fibroblasts, subsequent collagen deposition, and eventually leads to heart failure [11]. Recently, van Rooij, *et al.* [8] reported that miR-29b targets and inhibits a group of mRNAs that encode cardiac fibroblast proteins involved in fibrosis, and that the down-regulation of miR-29b after MI correlated with increased collagen types I and III, and fibrillin 1 in the peri-infarct and remote normal heart regions. Thus, we speculated that miR-29b overexpression might reduce heart tissue collagen and thereby lower the barrier to progenitor cell engraftment and survival. The aim of this study was to determine if miR-29b overexpression in the rat heart *in vivo* would effectively reduce barrier formation (collagen deposition) after MI and thereby enhance the efficacy of the iPSC-derived Tri-P based cell therapy in improving heart function after regional MI.

## Methods

### Laboratory animals

All research protocols conformed to the Guidelines for the Care and Use of Laboratory Animals published by the National Institutes of Health (National Academies Press, 8^th^ edition, 2011). All animal use protocols and methods of euthanasia (pentobarbital overdose followed by thoracotomy) used in this study were approved in this study were pre-approved by the University of Cincinnati Animal Care and Use Committee. An independent review and approval of our cell and virus methods was conducted by the Institutional Biosafety Committee (IBC).

## Experimental Designs and Methods

### In vitro study

To elucidate how miR-29b modulated molecular mechanisms involved in cardiac fibrosis modulated by assigning cardiac fibroblasts (CFb), the subjects were divided into the following treatment groups, 1) negative mimic (CFb^Ctrl^) served as control, 2) mimic microRNA-29b-1 (CFb^miR-29b^), or 3) miRCURY LNA™ microRNA-29b-1 inhibitor (CFb^Anti-29b^). After transfection all cell groups were placed in anaerobic glucose-free medium (Gibco, Invitrogen) and incubated in an anoxic chamber (*In Vivo* 500, Ruskin Life Science) for 3 hours in order to study the fibrosis related signaling pathway(s). Quantitative RT-PCR (qPCR) and Western blotting were performed to characterize miRNA expression and fibrosis-related gene expression as described below.

### Cell culture

Mouse iPSC were obtained from Dr. Shinya Yamanaka (Kyoto University, Japan) [6], [12], and maintained in Dulbecco's Modified Eagle's medium (DMEM) with 1000 IU/ml leukemia inhibitory factor (LIF, Chemicon, ESGRO), as described [12]. Mouse embryonic fibroblasts (MEF) obtained from embryos at 14 days post-coitum were prepared and treated with mitomycin-C (10 µg/mL) to control MEF overpopulation as previously described [12].

EB formation was promoted by placing iPSC in 25 µl hanging drops (∼250 cells per drop) and culturing the suspension in iPSC medium without LIF, then allowing cells to differentiate into large aggregates using differentiation media and growth factor conditions previously reported by Stevens et al. [13] to induce differentiation into cardiomyocytes. Cell culture conditions were further modified to optimize differentiation with lentiviral genomic manipulation.

Neonatal cardiomyocytes (neoCM) and CFb were prepared by selective attachment as described by Kusano et al. [14]. CFb and lentiviral vector using System Biosciences kit (SBI) - packaging HEK293FT cells (Invitrogen) were cultured in DMEM medium containing 10% FBS and antibiotics.

### Transfection of miRNA mimics and inhibitor

Cells were seeded (2.5×10^5^) in 6-well plates 24 h prior to transfection. The negative mimic, rno-miR-29b mimic (Dharmacon) or rno-miR-29b inhibitor (Exiqon) were added at the required final concentration (200 nmol/L for each well) after mixing with DharmaFECT Duo Transfection Reagent according to the manufacturer's instructions. The mixture was incubated for 20 minutes. The medium from each well was aspirated and replaced by 400 µL of the mixture and 1600 ul DMEM with 10% FBS. The transfection was performed in triplicate.

### Construction and production of lentiviral vectors

NCX1 promoter-driven luciferase signal is observed in the embryonic heart (E8.5–9.0), and sustained to the adult cardiomyocytes [15]. Elevated NCX1 expression during cardiomyocyte differentiation from ESCs [16] allowed NCX1 promoter-driven GFP signal to be a novel tool for monitoring cardiac differentiation of iPSC in *vivo*. iPSC^NCX1+^ were enriched by transduction with lentiviral vector encoding markers under the control of a cardiac promoter (NCX1), (kindly provided by Dr. Donald R. Menick) [15]. The final construct containing the cardiac specific promoter (NCX1) driving firefly luciferase (pLVX-NCX1-Fluc-PuroR-IRES-ZsGreen1) was constructed as described [6]. Endothelial cells (EC) derived from iPSC were enriched by transduction with lentiviral vector driven by EC specific promoter VE-cadherin, and the final vector construct was pCDH-VE-GFP [17]. Virus titers determined by p24-antigen Assay kit (Beckman Coulter, Fullerton, CA) as described [18]. The crude viral suspension was harvested from HEK293FT cell cultures 48 hours after transfection and filtered (0.45 µm) for use *in vitro*. Viral particles were stored at −80°C until use.

### Genetically selected iPSC^NCX1+^ and EC derived from iPSC

To construct an iPSC line overexpressing the NCX1 promoter or VE-cadherin promoter, iPSC (2.5×10^5^) seeded in Matrigel-coated 6-well plates were cultured in transduction medium following standard techniques [6], [17].

### Quantitative real-time PCR for miRNAs and fibrosis related gene expression

Total RNA was isolated by using Trizol reagent (Invitrogen), followed by DNAse treatment and purification using RNeasy mini column kit (Qiagen). cDNA was synthesized using miScript PCR Starter Kit (Qiagen) in a 20 µl reaction mixture. qPCR was performed on the iQ5 Gradient Real Time PCR system (Bio-Rad) using the protocol of miScript PCR Starter Kit (Qiagen). The primers for qPCR were synthesized as follows: **miR-29b1** primer: 5′-CGT AGC ACC ATT TGA AAT CAG TGT T-3′; reverse primer: 5′-GTG-CAG-GGT-CCG-AGG-T-3′; **TGF-β1** forward primer: 5′-AGG-GCT-ACC-ATG-CCA-ACT-TC-3′; reverse primer: 5′-CCA-CGT-AGT-AGA-CGA-TGG-GC-3′; **SDF-1α** forward primer: 5′-TAG-ACC-CCT-GAG-GAA-GGC-TG -3′, reverse primer: 5′- TGG-TGG-AGT-GTC-TTT-GTG-CT-3′; **MRTF-A** forward primer: 5′-GCC AAC CTG GAT GAC ATG AA-3′; reverse primer: 5′-CGC TCA ATC AGC TCT GTC TT-3′; **collagen I** forward primer: 5′-CTC TGT GCC TCA GAA GAA CT-3′; reverse primer: 5′-GGA ACC TTC GCT TCC ATA CT-3′; **collagen III** forward primer: 5′-CGG AAT TGC AGA GAC CTG AA-3′, reverse primer: 5′-CCA TCT TGC AGC CTT GGT TA-3′, **GAPDH** forward primer: 5′-CCA AGG CTG TGG GCA AGG TC-3′, reverse primer: 5′-GGC AGG TTT CTC CAG GCG G-3′. The relative expression level between treatments was then calculated using the following equation: relative gene expression = 2^−(ΔCt,sample-ΔCt,control)^.

### Western blotting

Cells were lysed with ice-cold cell lysis buffer plus protease inhibitor (Sigma). Protein samples (30 µg) were mixed and resolved in 4×SDS/PAGE sample buffer and boiled for 15 min before loading on 10% polyacrylamide gels (Precast Gels, ISC Bioexpress). The electrophoresed proteins were transferred from the gel to nitrocellulose membranes (Bio-Rad). Equal loading and transfer of proteins was confirmed by quantitative Ponceau red staining. The membranes were incubated for 60 minutes with 5% dry milk and Tris-buffered saline to block nonspecific binding sites. Membranes were immunoblotted overnight at 4°C with antibodies against p42/44 MAPK (pERK) (1∶1000, Cell Signal Technology), and actin (1∶2000, Santa Cruz) on a rocking platform. After three 5 min washings in Tris-buffered saline, the membranes were incubated for 60 min with HRP-conjugated secondary antibody, washed three times in Tris-buffered saline, and finally developed with the ECL plus kit (Bio-Rad).

### *In vivo* study

An ischemia-induced MI model was developed in 10–12 weeks old nude rats, Charles River), as previously described [4]. *In vivo* experimental design: negative mimic (Ctrl), miR-29b mimic (miR-29b), and miR-29b inhibitor (Anti-29b) were delivered into the rat hearts before MI as described previously [18]. A peritoneum based Tri-P including iPSC^NCX1+^, EC, and MEF was applied to the epicardial surface of the entire infarcted area 7 days after MI. Rats were assigned to experimental groups, as follows: 1) Sham operated rats had a loose suture placed around the left anterior descending (LAD) coronary artery (Sham group), 2) negative mimic pretreated rats with MI operation followed by Tri-P graft (Ctrl+MI+Tri-P), 3) miR-29b overexpression pretreated rats with MI operation followed by Tri-P graft (miR-29b+MI+Tri-P), 4) miR-29b knockdown pretreated rats with MI operation followed by Tri-P graft (Anti-29b+MI+Tri-P). Transthoracic echocardiography was performed at day 1 and at weeks 1, 2, 3, and 4 after cell patch application. To analyze the effect of anti-fibrosis treatment on heart function, additional infarcted hearts treated with negative mimic, or miR-29b mimic or miR-29b inhibitor were evaluated by echocardiography. Animals were euthanized at week 4 and hearts were fixed for fluorescent immunohistochemical assessment of angiomyogenesis. Additional hearts were perfused with MICROFIL-compound for 3-D vasculature analysis in a micro-CT scanner.

### Surgical procedures for miRNA administration in vivo and myocardial infarction

Nude rats (200–250 g) were anesthetized by spontaneous inhalation and maintained under general anesthesia with 1–2% isoflurane. The animals were mechanically ventilated using a rodent ventilator (Model 683, Harvard Apparatus) connected to a tracheal tube. Body temperature was maintained at 37°C throughout the surgical procedure. The thoracic cavity was then opened through a left side incision at the level of the 4^th^ intercostal space and the heart was exposed by a rib retractor. The aorta and pulmonary artery then were identified. A tiny 32 gauge catheter containing 50 µl of concentrated negative mimic, miR-29b mimic, or inhibitor (25 µM) was advanced from the apex of the left ventricle to the aortic root. The aorta and pulmonary arteries were clamped distal to the site of the catheter and the solution was injected. The clamp was maintained for 10 seconds during which the heart pumped against a closed system. This procedure allows the solution containing the lentivirus to perfuse the coronary arteries and the myocardium. After 10 seconds, clamps on the aorta and pulmonary artery were released and the chest was closed as described [19], [20].

The left anterior descending artery (LAD) was then ligated permanently with a 6-0 polyester suture 1 mm from the tip of the normally positioned left auricle to induce an infarcted area in the left ventricular anterior wall. Before closing the thoracic cavity, positive end-expiratory pressure was applied to fully inflate the lungs, and then muscle layers and skin were closed separately. 7 days after MI, the left thoracic cavity was re-opened. The cell-seeded side of the substrate biomaterial (peritoneum) was apposed to the surface of LAD distribution overarching the MI area followed by chest closure.

Rats were anesthetized by intraperitoneal injection of pentobarbital (100 mg/kg body weight), and the adequacy of anesthesia was evaluated by monitoring hind limb reflexes. When unconscious state was induced, the hearts were excised from the thorax at the ascending aorta and rinsed with PBS.

### Tri-cell based peritoneum preparation

To harvest the substrate allograft biomaterial (peritoneum), the abdominal cavity of additional Sprague Dawley (SD) rats (200–250 g, Charles River) was opened and abdominal contents were reflected to the left side to expose the peritoneum.

A small diameter ring was inserted behind the peritoneum under the detached portion of the substrate. This ring was then covered with a larger ring on the surface of peritoneum. After rinsing blood from the harvested disc of peritoneum (diameter = 20 mm) with PBS, the peritoneum patches were placed in a cell culture dish with MEF-medium at 37°C in a humidified atmosphere of 95% room air: 5% CO2.

iPSC^NCX1+^ and EC as well as MEF were cultured onto the peritoneum (1∶1∶2) in CF-medium at 37°C in a humidified atmosphere of 95% room air: 5% CO_2_ for 7 days [6].

### Analysis of left ventricular fibrotic area and anterior wall thickness (AWT)

Fixed heart tissues embedded in paraffin and LV cross-sections from the mid-LV to apex stained with Masson's Trichrome were used to quantify fibrosis in the left ventricles. An Olympus BX41 microscope equipped with CCD (Magna-Fire TM, Olympus) camera captured LV area images on each slide. LV fibrotic area and total LV area of each image were measured using the Image-Pro-Plus program (Media Cybernetics Inc.), and the fibrotic area was reported as a percentage of the total LV area [6].

### Histology and immunohistochemistry

After fixation in 4% paraformaldehyde PBS, heart specimens (n = 6 for each group) from rats 4 weeks after Tri-P graft were embedded in OCT, frozen in liquid nitrogen, and then sectioned at 8 µm thickness. GFP expression was used to identify transplanted cell migration and new vessel formation. Cardiac troponin T antibody (cTnT) (Thermo) was used to evaluate cardiac myogenesis; and 4′, 6-diamino-2-phenylindole (DAPI, Sigma, St. Louis, MO) was used to identify nuclei. Secondary antibodies were purchased from Jackson Laboratories. Fluorescent imaging was performed with an Olympus BX41 microscope (Olympus America Inc.) equipped with epifluorescence attachment and images were recorded using a digital camera with MagnaFire™ 2.1 software.

### Echocardiography

Transthoracic echocardiography (iE33 Ultrasound System, Phillips, Andover, MA) with a 15-MHz probe was performed to analyze unstressed heart function [4]. Hearts were imaged in 2-D long-axis view at the level of the greatest LV diameter with animals under light general anesthesia. This view was used to position the M-mode cursor perpendicular to the LV anterior and posterior walls. LV end-diastolic and end-systolic diameters were measured from M-mode recordings. LV ejection fraction (EF) was calculated as: EF (%) = [left ventricular end-diastolic dimension (LVDd)^3^ minus left ventricular end systolic dimension (LVDs)^3^/(LVDd)^3^]×100. LV fractional shortening (FS) was also determined as [(LVDd−LVDs)/LVDd]×100. Anterior wall end-diastolic and end-systolic thickness was measured using data from short-axis. All measurements were performed according to the American Society for Echocardiology leading-edge technique standards, and averaged over three consecutive cardiac cycles.

### Micro computed tomography imaging

In general, 5 weeks after MI, the animals were deeply anesthetized (ketamine/xylazine 100 mg/kg//10 mg/kg) and heparinized with heparin saline (100 IU/Kg). The thoracic cage was opened and the thoracic aorta isolated, after which a polyethylene cannula was inserted. The arterial cannula was connected to a syringe pump and heparin infused at constant pressure and flow (rate of 200 µl/min); before perfusion, the superior vena cava and inferior vena cava (IVC) were opened as vents. All animals were euthanized with i.v. KCl solution (25 mmol/l), to stop the heart in diastole. Then, MICROFIL silicone rubber including 8 ml MV-compound (MICROFIL MV-122, Flow Tech), 10 ml MV-diluents and 5% MV curing agent, was retrogradely infused into the coronary arteries via the thoracic aorta. The MICROFIL compound perfusion was continued until the material flowed freely from the IVC. The heart then underwent a Micro-CT scan on an Inveon Multimodality System (Siemens). Using Inveon workstation and vendor software, we performed image segmentation to define the Tri-P and its blood vessels followed by quantitative analysis of blood vessel volume within the patch and blood vessel number. These parameters allowed us to make direct comparisons across different groups and to evaluate the effectiveness of various treatments.

### Statistical analysis

Results were statistically analyzed with the use of the StatView 5.0 software package (Abacus Concepts Inc., Berkeley, CA). All values are expressed as mean ± S.E.M. Unpaired t-test was applied as appropriate for comparison between two treatment groups. One-way ANOVA, using the post-hoc Bonferroni/Dunn test was performed for comparisons of multiple groups in each of the specific experimental designs presented in the figures with multiple treatment groups. Two-way ANOVA was used to evaluate the effects within and between groups over time. Two-way ANOVA for repeated measurements was performed for echocardiographic A p-value<0.05 is considered statistically significant.

## Results

### *In vitro* study

#### Collagen synthesis is mediated by miR-29b in the heart fibroblasts

**Figure 1:**
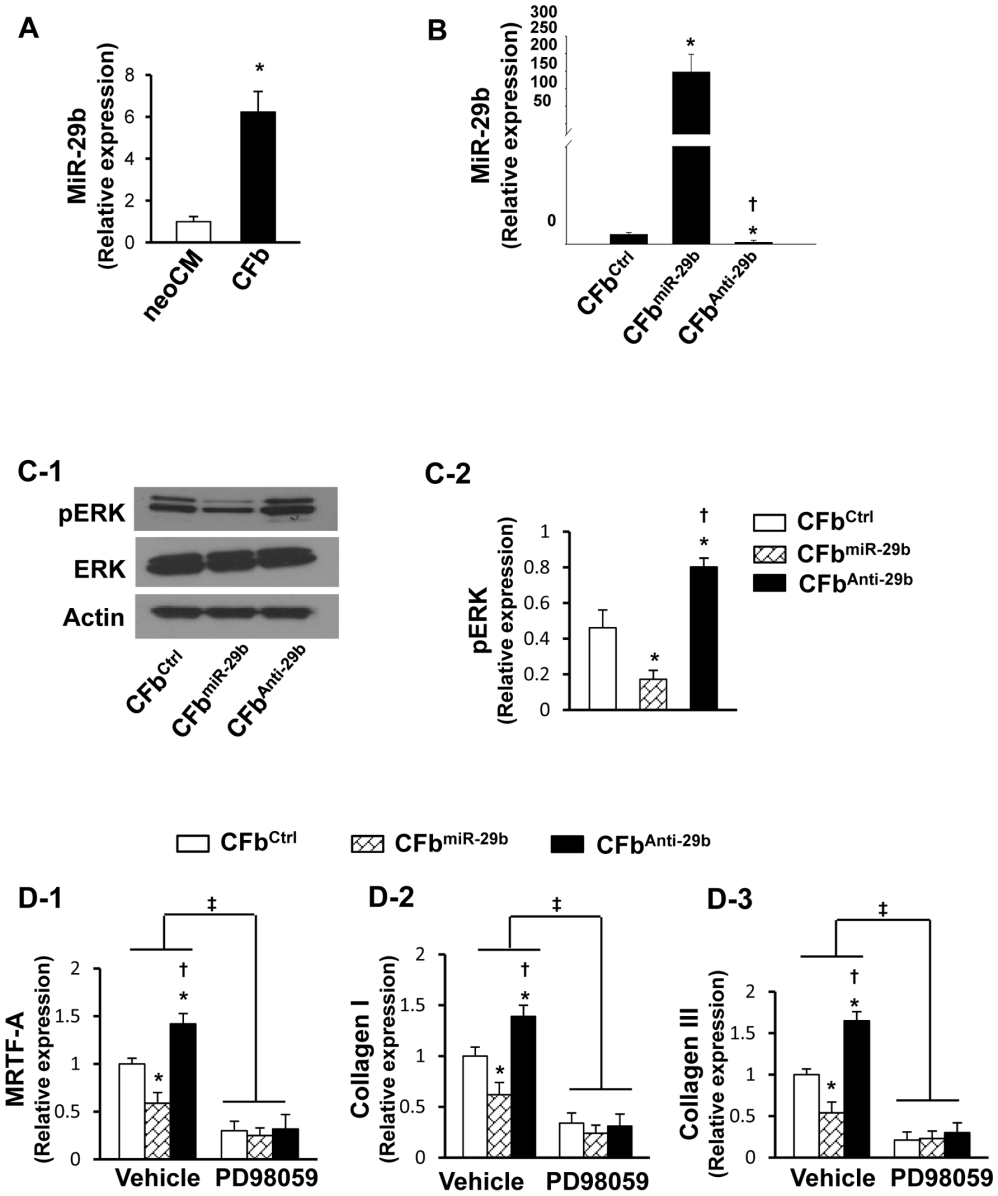
Effect of miR-29b overexpression *in vitro* on cardiac fibrosis. (A): Quantitative RT-PCR (qPCR) analysis for miR-29b expression in neonatal cardiomyocytes (neoCM) and cardiac fibroblasts (CFb). *p<0.05 vs. neoCM. All values expressed as mean ± SEM. n = 4 for each group. (B): Transfection efficiency identified by qPCR in cardiac fibroblasts following transfection with control mimic (CFb^Ctrl^), miR-29b1 mimic (CFb^miR-29b^), or miR-29b1 inhibitor (CFb^Anti-29b^), respectively. *p<0.05 vs. CFb^Ctrl^; ^†^p<0.05 vs. CFb^miR-29b^. All values expressed as mean ± SEM. n = 4 for each group. (C): Western blot analysis for the mechanism of fibrosis in CFb under anoxic condition (C-1) Quantitative data for pERK (C-2). p-ERK, extracellular signal-regulated kinase phosphorylation; ERK, extracellular signal-regulated kinase. *p<0.05 vs. CFb^Ctrl^; ^†^p<0.05 vs. CFb^miR-29b^. All values expressed as mean ± SEM. n = 4 for each group. (D): qPCR analysis for the mechanism of fibrosis in CFb under anoxic condition. Quantitative data for expression of MRTF-A (myocardin-related transcription factor A) (D-1), collagen I (D-2), and collagen III (D-3) in various experimental groups after treatment with either vehicle or PD98059. PD98059 is an inhibitor of ERK-MAPK signaling pathway. *p<0.05 vs. CFb^Ctrl^; ^†^p<0.05 vs. CFb^miR-29b^; ^‡^p<0.05 vs. vehicle group. All values expressed as mean ± SEM. n = 8 for each group.

**Figure 2:**
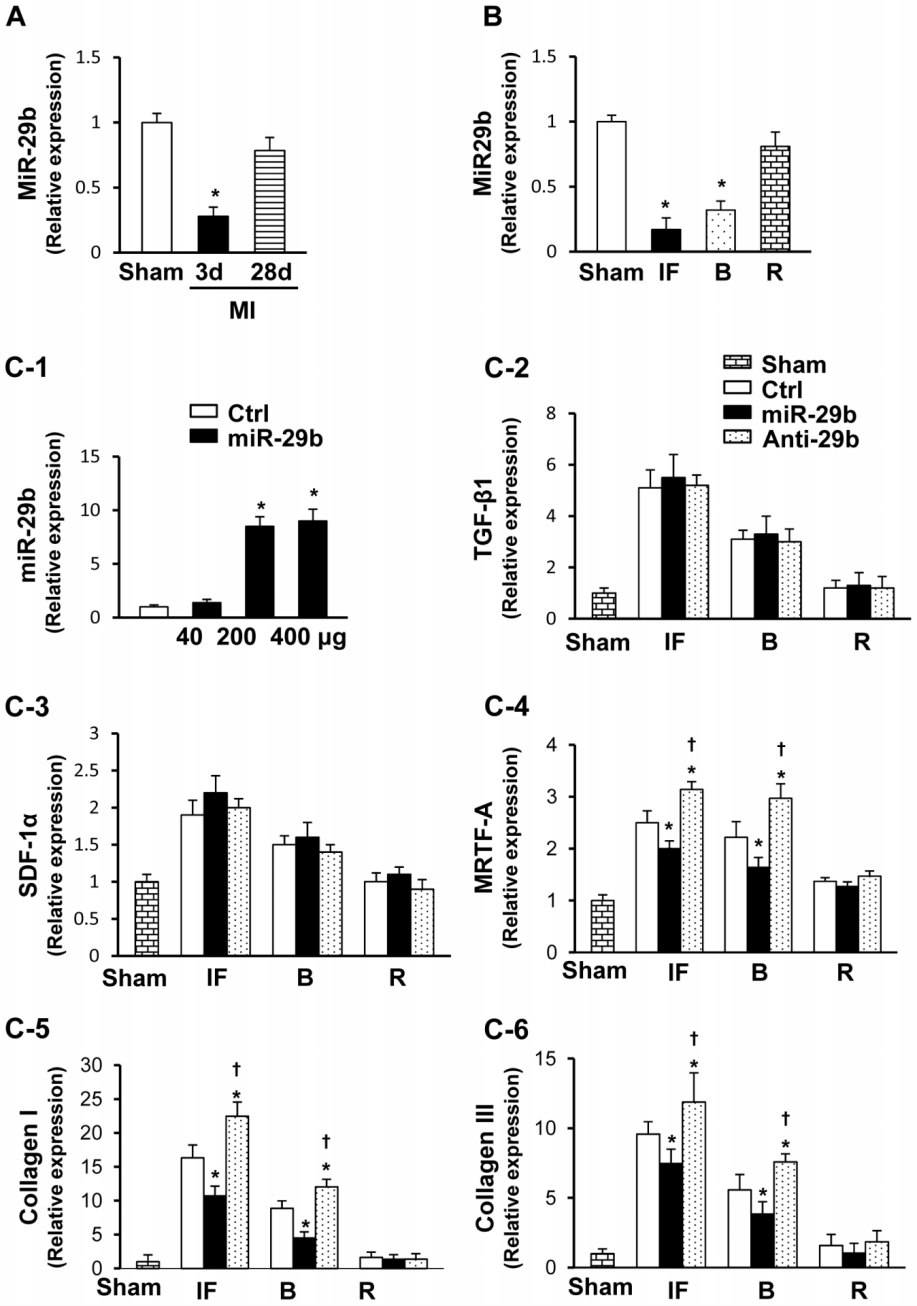
Effect of miR-29b *in vivo* on cardiac fibrotic gene related expression. (A): qPCR analysis for miR-29b expression in infarcted heart at 3 days or 28 days. *p<0.05 vs. Sham group. All values expressed as mean ± SEM. n = 3 for each group. (B): qPCR analysis for miR-29b expression in infarcted area, border area, and remote area 3 days post-MI, respectively. Sham, sham operated group with loose suture around LAD; MI, myocardial infarction; IF, infarcted area; B, border area; R, remote area. *p<0.05 vs. Sham group. All values expressed as mean ± SEM. n = 3 for each group. (C-1): qPCR analysis of miR-29b expression in heart after *in vivo* administration. Quantitative data for fibrosis related genes including exprression of TGF-β1 (C-2), SDF-1α (C-3), MRTF-A (C-4) collagen I (C-5), and collagen III (C-6) in infarcted heart 3 days after intramyocardial gene delivery of control mimic (Ctrl), miR-29b1 mimic (miR-29b), or miR-29b1 inhibitor (Anti-29b). *p<0.05 vs. CFb^Ctrl^; ^†^p<0.05 vs. CFb^miR-29b^. All values expressed as mean ± SEM. n = 4 for each group.

**Figure 3:**
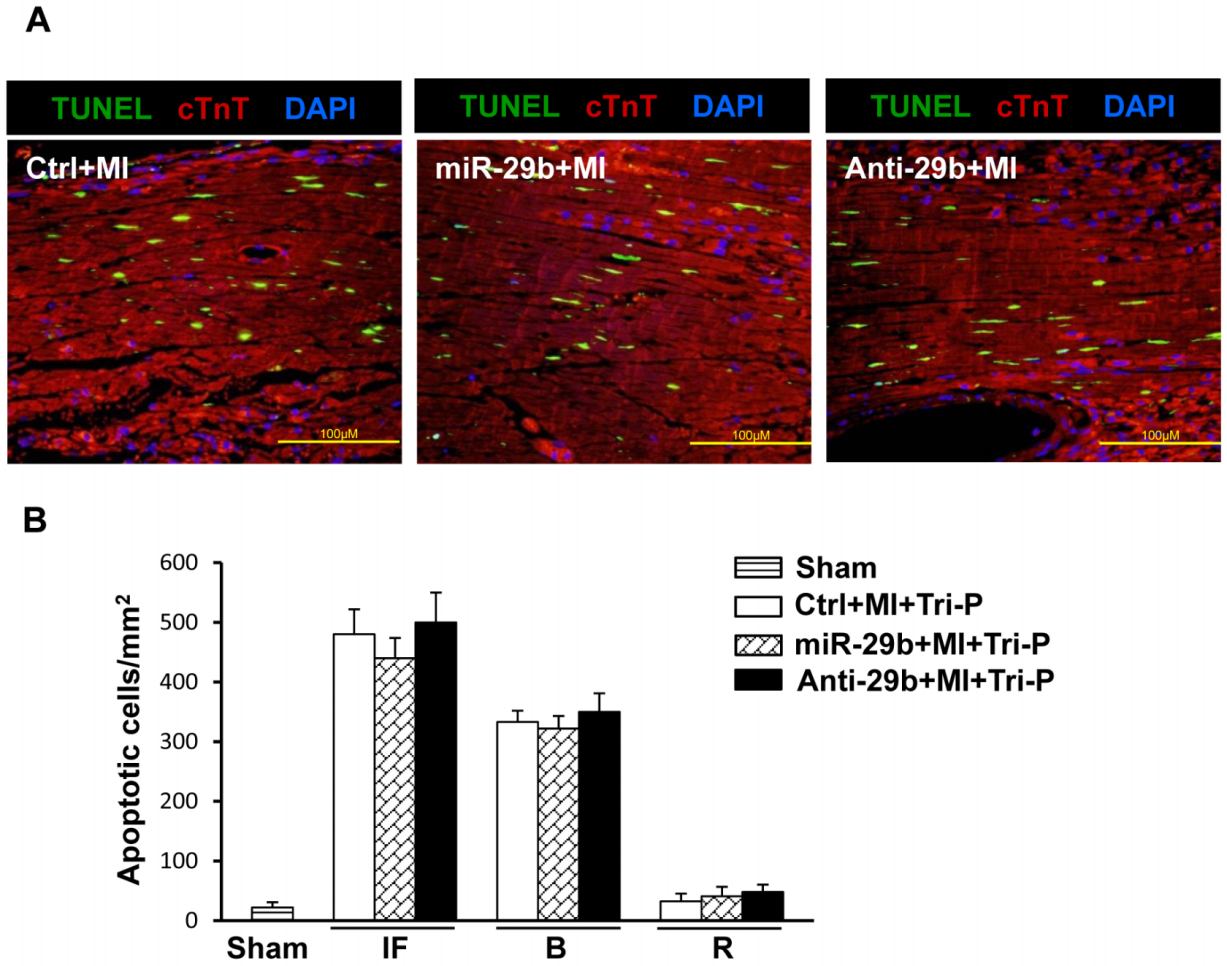
Effect of miR-29b *in vivo* on cardiomyocyte apoptosis. (A): TUNEL staining for cardiomyocyte apoptosis in the infarcted heart 3 days after MI. Scale bar = 100 µm. Original magnification, ×400.*p<0.05 vs. Ctrl; ^†^p<0.05 vs. miR-29b group. Sham, sham operated group with loose suture around LAD; Ctrl, intramyocardial gene delivery with control; miR-29b, intramyocardial gene delivery with miR-29b-1 mimic; Anti-29b, intramyocardial gene delivery with miR-29b-1inhibitor. All values expressed as mean ± SEM. n = 4 for each group. (B): Quantitative data for numbers of TUNEL positive nuclei in the infarcted area, border area, and non-ischemic remote area in various groups 3 days after MI. Sham, sham operated group with loose suture around LAD; Ctrl, intramyocardial gene delivery with control mimic; miR-29b, intramyocardial gene delivery with miR-29b-1 mimic; Anti-29b, intramyocardial gene delivery with miR-29b-1inhibitor. All values expressed as mean ± SEM. n = 6 for each group.

MiR-29b has been reported to directly target mRNAs that encode various ECM proteins involved in fibrosis [3]. To analyze the role of miR-29b on cardiac fibrosis, the total mRNA of rat neonatal cardiomyocytes (neoCM) and CFb were isolated respectively, and qPCR revealed that miR-29b was expressed significantly more in CFb than in neoCM (p<0.05, Fig. 1A). To further study the molecular signaling pathway involved in fibrosis, CFb were transfected with negative mimic (CFb^Ctrl^), miR-29b mimic (CFb^miR-29b^), or miR-29b inhibitor (CFb^Anti-29b^). Quantitative RT-PCR showed that the expression of miR-29b was significantly increased in CFb^miR-29b^ group (p<0.01), and was significantly reduced in CFb^Anti-29b^ group as compared with CFb^Ctrl^ group (p<0.05, Fig. 1B). Under normoxia, there was no difference in phosphorylation of ERK (pERK, p42/44MAPK) expression detected among these three cell groups (data not shown). However, under anoxia CFb overexpressing miR-29b (CFb^miR-29b^) exhibited a significant decrease (p<0.05) in pERK (Fig. 1C1–2), myocardin-related transcription factor-A (MRTF-A, Fig. 1D-1), collagen type I (Fig. 1D-2), and collagen type III (Fig. 1D-3). These suppressive effects were reversed in miR-29b knockdown cells (CFb^Anti-29b^), which exhibited significant increases in pERK, MRTF-A, collagen I, and III levels (p<0.05). Importantly, the effect of miR-29b overexpression on the fibrotic related genes MRTF-A, collagen I, and collagen III was abolished in CFb in presence of the selective inhibitor of pERK, PD98059 (Fig. 1D1–3).

To further explore the *in vivo* findings, several regions of the infarcted rat hearts including the infarcted region (IF), border region (B) and remote region (R) were harvested to analyze the effect of miR-29b overexpression or knockdown on cardiac fibrosis. Compared with sham operated hearts (Sham group), a significant down-regulation of miR-29b was observed in the hearts 3 days post-MI (p<0.05) (Fig. 2A), which was most prominent in the infarcted area (Fig. 2B). 4 weeks post-MI, slight down-regulation of miR-29b was detected in the infarct areas, but was not statistically different from the Sham group (Fig. 2A). To further analyze whether miR-29b overexpression interfered with post-MI collagen deposition *in vivo*, intramyocardial gene delivery was performed (Fig. 2C-1). Although no significant differences of TGF-β1 (Fig. 2C-2) and SDF-1α (Fig. 2C-3) levels were observed in miR-29b pretreated heart, miR-29b pretreatment significantly decreased the level of MRTF-A, collagen I and III (p<0.05) when compared to the Ctrl group in the infarcted and border areas. Conversely, when the heart had been pretreated with Anti-29b, the opposite effects were observed (Fig. 2C4–6). There was no statistically significant difference detected in MRTF-A, collagen I and III expression in the remote normal myocardium among these groups.

During the early time points of MI, the observed decline in LV function in the regionally infarcted heart may primarily reflect the loss of cardiomyocytes [18]. To evaluate the role of miR-29b on cardioprotection, apoptotic cardiomyocytes were assessed 3 days after MI (Fig. 3). However, no obvious anti-apoptosis effect of miR-29b overexpression was observed in comparison to Ctrl group and Anti-29b group.

#### miR-29b-mediated fibrotic size facilitates mobilization of iPSC^NCX1+^ and angiomyogenesis

**Figure 4:**
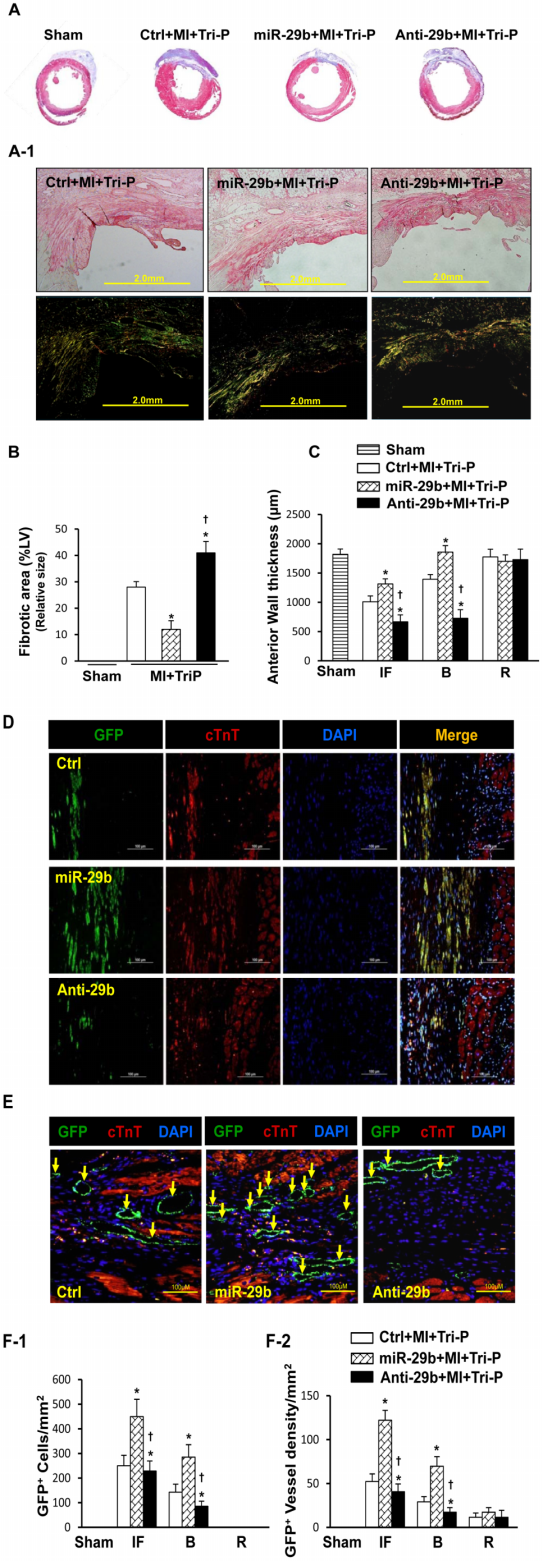
Effect of miR-29b on fibrotic size, iPSC migration, and angiogenesis in the infarcted heart 4 weeks after Tri-P graft. (A): Masson's Trichrome staining for fibrotic size in various treatment groups. (A-1): Picrosirius red staining of fibrosis under polarized light in various treatment groups. (B): Quantitative data for LV fibrotic size in various treatment groups. *p<0.05 vs. Ctrl+MI+Tri-P; ^†^p<0.05 vs. miR-29b+MI+Tri-P group. All values expressed as mean ± SEM. n = 6 for each group. (C): Quantitative data for LV anterior wall thickness (AWT) in various treatment groups. *p<0.05 vs. Ctrl+MI+Tri-P; ^†^p<0.05 vs. miR-29b+MI+Tri-P group. All values expressed as mean ± SEM. n = 6 for each group. (D): Immunofluorescence microscopy showed a cluster of GFP positive iPSC migrated from Tri-P into the infarcted area (green color), cardiomyocytes were confirmed by cTnT staining. Color codes given at the top. Scale bar = 100 µm. Original magnification, ×200. (E): Immunofluorescence microscopy for angiogenesis in the infarcted heart after Tri-P graft, identified by GFP expression (green color, arrows). Color codes given at the top. Scale bar = 100 µm. Original magnification, ×400. (Left panel = Ctrl group; middle panel = miR-29b; right panel = Anti-29b. (F): Quantitative data for angiogenesis in the infarcted area, border area, and non-ischemic remote area in various groups. (F-1): The number of GFP positive cells was significantly higher in miR-29b group than Ctrl or Anti-29b group. (F-2): The number of GFP positive vessels in various treatment group. *p<0.05 vs. Ctrl+MI+Tri-P; ^†^p<0.05 vs. miR-29b+MI+Tri-P group. Sham, sham operated group with loose suture around LAD; Ctrl, control mimic pretreatd rat with Tri-cell patch graft; miR-29b, miR-29b mimic pretreaed rat with Tri-cell patch graft; Anti-29b, miR-29b inhibitor pretreated rat with Tri-cell patch graft. All values expressed as mean ± SEM. n = 6 for each group.

Rats were subjected to LAD ligation for 7 days, followed by apposition of the Tri-P to the infarcted heart region. LV fibrosis, analyzed by Masson's Trichrome staining 4 weeks after patch transplantation, was significantly reduced in the miR-29b pretreated group. This was accompanied with significant increase of anterior wall thickness (AWT) (Fig. 4A–C) as compared with the Ctrl group. This benefit was abolished in the Anti-29b pretreated group (Fig. 4A,B and C), as indicated by a significantly larger infarct size and decreased AWT (p<0.05). The anti-fibrosis effect of miR-29b pretreatment was further supported by picrosirius red staining under polarized light, which showed significantly lower levels of cardiac fibrosis 4 weeks after Tri-P graft (Fig. 4A-1). To determine the relationship between collagen deposition and iPSC^NCX1+^ migration post-MI, additional heart tissues were used for immunostaining analysis 4 weeks after Tri-P transplantation. These showed uniform GFP^+^ cells (green color, Fig. 4D) with some differentiation into cardiomyocytes, confirmed by positive cardiac troponin T (cTnT) staining, a specific cardiomyocyte marker (Fig. 4D). In the miR-29b pretreated group, significantly more GFP^+^ cells migrated into the infarcted area as compared with Ctrl. However, very few GFP^+^ cells were observed in the Anti-29b pretreated groups, *p<0.05 (Fig. 4F-1). In the Anti-29b group, the infarct size was significantly increased as compared with Ctrl and the miR-29b pretreated group. Furthermore, an increased number of newly formed blood vessels, identified by GFP expression, were detected in miR-29b pretreated hearts compared with the other two groups 4 weeks after Tri-P application (Fig. 4E and 4F-2). The number of vessels in the Anti-29b group was significantly lower than in the other two groups.

#### Three-dimensional micro-CT image for neovascularization after Tri-P transplantation

**Figure 5:**
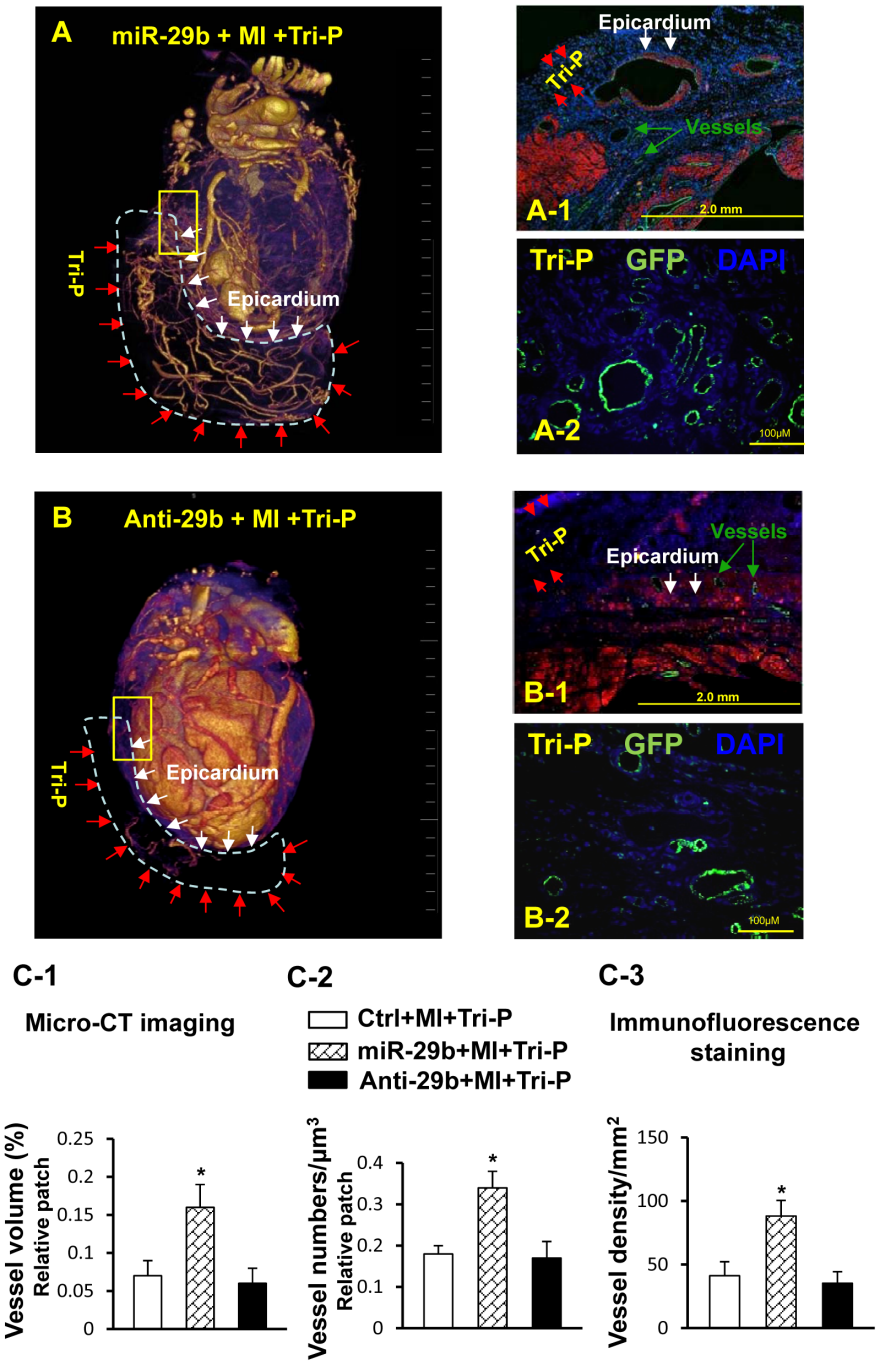
Collateral circulation from the cell patch to the native coronary arteries. Micro-CT imaging for collateral circulation formation in miR-29b+MI+Tri-P (A) and Anti-29b+MI+Tri-P group (B). Prominent collateral circulation from the cell patch to the native coronary arteries was observed in miR-29b pretreated rat as compared to Anti-29b pretreated rat. Characteristics of Tri-P in miR-29b+MI+Tri-P group (A-1) and Anti-29b+MI+Tri-P group (B-1). Fluorescence microscopy showed that Tri-P was applied to the LV epicardium of the infarcted area at 4 weeks after Tri-P implantation (data in other groups not shown). Fluorescence microscopy demonstrated newly formed vessels confirmed by GFP antibody (green color) in the cell patch of miR-29b+MI+Tri-P group (A-2) and Anti-29b+MI+Tri-P group (B-2). (C-1): Quantitative data for micro-CT 3D images, including vessel volume (%) and (C-2): vessel numbers/µm^3^ in Tri-P. *p<0.05 vs. Ctrl+MI+Tri-P group. (C-3): Quantitative data for vessel density visualized by GFP expression in Tri-P. miR-29b, miR-29b mimic pretreated rat with Tri-cell patch graft; Anti-29b, miR-29b inhibitor pretreated rat with Tri-cell patch graft. *p<0.05 vs. Ctrl+MI+Tri-P group. All values expressed as mean ± SEM. n = 6 for each group.

Three-dimensional (3-D) architecture and functional connectivity were evaluated by micro-CT at 4 weeks after Tri-P graft. The 2-D structure of collateral vessels passing from the graft to the coronary circulation was visualized clearly by micro-CT imaging (Fig. 5), 3-D micro-CT video imaging was attached. Additionally, histological analysis of the 2-D architecture of the vessels, performed on the same tissues after micro-CT scanning, revealed that the new vessels had incorporated transplanted cells from the cell patch (Fig. 5A and B). Although Tri-P transplantation induced a robust neo-vascularization as early as 1 week post-patch graft, no differences in vessel volume or vessel number were detected between the miR-29b pretreated group and the Anti-29b pretreated group at that early time point (data not shown). However, a complex vascular network was detected in the cell patch at 4 weeks after patch transplantation, which was collateral with native coronary arteries (Fig. 5A) and exhibited greater vessel volume and vessel number (Fig. 5C-1), the hallmarks of the dynamic angiogenic process that was much more robust in the miR-29b pretreated group than in other groups (Fig. 5C-2). Furthermore, GFP positive labeling (Fig. 5C-3) confirmed significant vascularization in the Tri-P region in the miR-29b pretreated group.

#### Echocardiography findings

**Figure 6:**
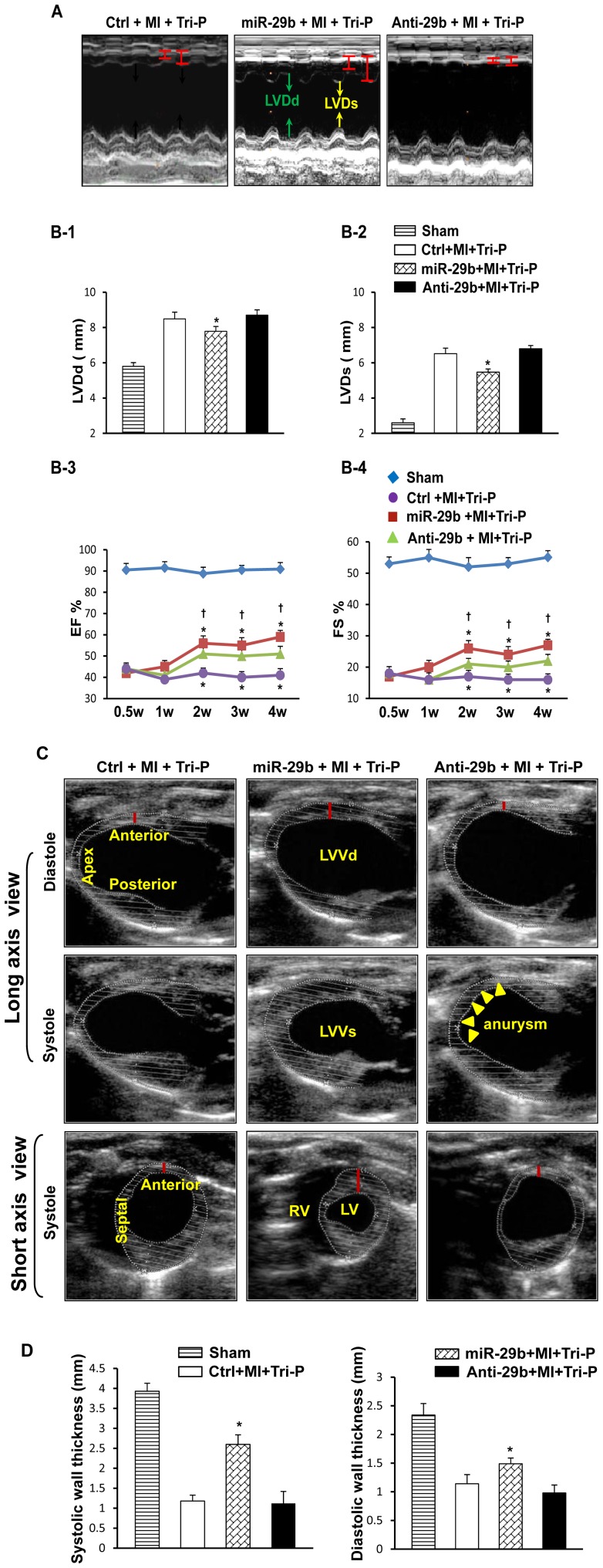
Cardiac function assessed by echocardiography 4 weeks after Tri-P implantation. (A): M-mode echocardiograph data in three groups. (B): Quantification analysis for heart function. Quantitative data for LVDd (B-1), LVDs (B-2), EF (B-3), and FS (B-4) 4 weeks after Tri-P implantation. *p<0.05 vs. Ctrl+MI+Tri-P group; ^†^p<0.05 vs. Anti-29b+MI+Tri-P group. LVDd, left ventricular end-diastolic diameters; LVDs, left ventricular end-systolic diameters; EF, ejection fraction index; FS, fractional shortening. All values expressed as mean ± SEM. n = 6 for each group. (C): Two-D mode echocardiograph data in three groups, analyzed by long-axis and short-axis views. *p<0.05 vs. Ctrl+MI+Tri-P group; ^†^p<0.05 vs. miR-29b+MI+Tri-P group. Ctrl, control mimic pretreatd rat with Tri-cell patch graft; miR-29b, miR-29b mimic pretreated rat with Tri-cell patch graft; Anti-29b, miR-29b inhibitor pretreated rat with Tri-cell patch graft. White dotted lines indicate endocardium and epicardium. (D): Quantification analysis for systolic (left) and diastolic (right) free wall thickness. *p<0.05 vs. Ctrl+MI+Tri-P group. All values expressed as mean ± SEM. n = 6 for each group.

2D-mode echocardiography (Fig. 6A) detected that LV remodeling was greatly improved in miR-29b pretreated rats as indicated by significant reductions in LVDd and LVDs 4 weeks after Tri-P transplantation when compared with the other two groups (Fig. 6B1–2), however, the heart rate of each group did not show any difference (Table 1). LV function, assessed as ejection fraction (EF), showed no obvious changes at 1 week after Tri-P placement. However, EF was significantly increased at 2 weeks in miR-29b pretreated hearts as compared with Ctrl (Fig. 6B-3). A similar trend in fractional shortening (FS) was also observed in miR-29b pretreated hearts as compared with Ctrl. However, the Anti-29b group did not show any significant EF and FS improvement with time (Fig. 6B3–4). Ventricular remodeling in the Ctrl group was evident, as indicated by reduction in LV anterior wall thickness (Fig. 6C). In contrast, LV anterior wall thickness and global cardiac LV performance were significantly increased in the miR-29b pretreated group 4 weeks after Tri-P implantation as compared with Ctrl group. LV remodeling was severe in the Anti-29b group, as indicated by significant reduction of LV anterior wall thickness (p<0.05) and a LV aneurysm was seen in some hearts in this group. Histological analysis and micro-CT imaging supported these notable *in vivo* echocardiography data. However, no obvious heart functional changes were observed in miR-29b alone treatment group (data not shown).

**Table 1:** Heart rate (bpm) changes measured by echocardiography.

Group	Before MI	MI	MI+Tri-P				
			0.5w	1w	2w	3w	4w
Ctrl+MI+Tri-P	249±20	259±30	249±19	239±35	242±31	239±29	260±18
MiR-29b+MI+Tri-P	242±25	251±23	259±22	253±29	249±22	251±28	259±29
Anti-29b+MI+Tri-P	251±22	260±19	260±20	251±30	249±33	239±32	259±30

The heart rate of Sham group was 259±30 bpm (beat per minute).

Ctrl+MI+Tri-P, control pretreated rat with Tri-cell patch graft; miR-29b+MI+Tri-P, miR-29b mimic pretreated rat with Tri-cell patch graft; Anti-29b+MI+Tri-P, miR-29b inhibitor pretreated rat with Tri-cell patch graft. All values were expressed as mean ± SEM. n = 6 for each group.

## Discussion

Cardiac tissue engineering is developing into a promising alternative to surgical and pharmacological approaches for effective treatment of ischemic heart diseases. Such cellular and tissue engineering can overcome the limitations of conventional treatment options including limited myocardial regeneration and lack of donor organs for transplantation. To be clinically useful, such cellular technologies must permit the construction of functional cardiac tissue grafts with physiological properties similar to native myocardium as well as sustainable viability after transplantation. Therefore, it is important to develop a pre-vascularized cell patch to supply sufficient oxygen and nutrients, allowing for enhanced transplanted stem/progenitor cell survival. However, revascularization in the myocardial infarct region by itself is not sufficient to restore systolic function post-MI [21]. Recently, we have reported the strategies to engineer a natural peritoneum-based vascularized cell sheet [6] with three types of cells (Tri-P) including iPSC^NCX1+^, EC and MEF. Importantly, a dynamic angiogenic process was observed in the Tri-P with a very dense arterial network that connected to the native coronary circulation confirmed by micro-CT imaging. These new vessels create a favorable microenvironment for transplanted progenitor cells. This process led to enhanced capillary formation, higher progenitor cell survival and subsequent LV function improvement after Tri-P implantation in a MI model.

Cardiac fibroblasts are critical for maintenance of cardiac structural integrity and are also involved in stem/progenitor cell homing and migration into the infarcted heart via dynamic regulation of ECM. However, the mechanisms involved in cardiac fibroblast activation and fibrosis remain unknown. The results of the present study demonstrate a distinctive role for miR-29b-mediated collagen deposition in the animal model of acute MI followed by iPSC derived Tri-P for the repair of myocardial infarction. There are three major findings of this study: 1) Overexpression of miR-29b, by blocking the activation of the p42/44 MAPK-MRTF signaling pathway, inhibits myofibroblast formation and attenuates collagen synthesis. 2) Reduced collagen deposition associated with miR-29b overexpression in scar tissue after MI facilitates iPSC^NCX1+^ penetration from the Tri-P into the infarcted area, and results in restoration of LV function after MI. 3) Overexpression of miR-29b enhances new vessel formation from the cell patch and these vessels connect to the native coronary circulation.

Fibrosis post-MI, not only increases risk of adverse cardiovascular events such as ventricular dysfunction and arrhythmia, but also creates a barrier that limits transplanted progenitor cells' migration, engraftment, survival, and associated functional benefits. Recently, altered expression of miRNAs has been shown to mediate myocardial fibrosis post-MI [3], [8], which suggest that use of miR-29b, as a fibroblast-enriched miRNA [11], is a potential anti-fibrosis target therapy based on its ability to directly target mRNAs that encode ECM proteins involved in fibrosis. To probe the miR-29b-triggered downstream signal pathway involved in cardiac fibrosis *in vitro*, we isolated cardiac fibroblasts and transfected them with negative mimic (Ctrl), or miR-29b mimic (miR-29b), or miR-29b inhibitor (Anti-29b). We discovered that overexpression of miR-29b significantly down-regulated collagen synthesis during anoxia through inhibited pERK activity as well as MRTF-A, a serum response factor (SRF) serving as a cofactor that responds to TGF-β in fibroblasts, contributing to SMA-enriched myofibroblasts [22]. Furthermore, other ECM proteins including Elastin and fibrillin 1, as the predicted targets of miR-29b, were significantly down-regulated in miR-29b overexpressing group (data not shown). This favorable anti-fibrotic effect of miR-29b on fibrosis was reversed by the pERK inhibitor, PD98059, suggesting the crucial role of ERK signaling pathway in fibroblast proliferation, and myofibroblast differentiation [2], [23], [24]. Our *in vivo* findings demonstrate that the post-MI fibrotic processes can be reversed by miR-29b intramyocardial delivery leading to decreased synthesis of MRTF-A, collagen I and III in the infarcted/border regions. The pretreatment strategy of intramyocardial delivery with miR-29b followed by Tri-P, in the setting of MI, resulted in restoration of LV mechanical function after MI. With regard to the function in cardiac fibroblasts, miR-29b may attenuate scar barriers to progenitor cell infiltration thereby facilitating iPSC^NCX1+^ penetration from the Tri-P into the infarcted area. Cardiac fibroblasts, the most numerous non-cardiomyocyte cell populations in heart tissue, generate essential autocrine/paracrine factors to maintain the functional integrity of the myocardium [25], [26], [27], [28]. Since miRNAs target not only single genes but also functionally related gene networks, the paracrine effect of miR-29b overexpressing fibroblasts may contribute to increased iPSC^NCX1+^ migration and survival.

For vascular network analysis, the classical methods of assessment such as LASER Doppler, magnetic resonance angiography, or positron emission tomography are not quantitative and are limited by 2-D imaging or provide only superficial blood flow data [29]. However, micro-CT using silicone rubber emulsions [30] presents a uniquely promising imaging modality for vascular exploration [31], which provides a high resolution 3-D volumetric representation for visualizing vascular networks and permitting direct assessment of the level of angiogenesis or neo-vascularization [32]. By micro-CT analysis, we demonstrated collateral blood vessel network formation arose from the cell patch and connected to native coronary arteries as a prominent feature in miR-29b pretreated hearts in contrast to the Anti-29b group. This vascular origin was confirmed by GFP expression.

Since prospective applications of the Tri-P graft may be directed at coronary artery syndromes and ischemic heart disease, challenges exist in assuring transplanted progenitor cells survive in ischemic microenvironments where oxygen and nutrient supply is severely compromised. Therefore, it is extremely important to promote concomitant angiogenesis providing sufficient blood flow, oxygen, and nutrient substrates, while moving metabolic products away from the ischemic myocardium with transplanted progenitor cells [33].

In the current study, we observed a significantly increased number of GFP^+^ myocytes (identified by cTnT staining) in the infarcted region of the miR-29b pretreated group. Furthermore, the differences in micro-CT imaging and number of GFP^+^ cells among the study groups were consistent and positively correlated with the differences measured in LV function. For the first time, in contrast to the untreated infarcted heart, angiogenesis or neovessel formation in the Tri-P integrated with the coronary arteries, which can potentially serve as a blood supply to the underlying ischemic myocardium. Taken together, these findings suggest that miR-29b and its downstream target genes, as supplements to cell-based therapies, serve as a promising therapeutic regulator to retard, limit or reverse fibrosis post-MI. miR-29 overexpression in combination with the Tri-P may facilitate iPSC^NCX1+^ migration and survival, as well as permit neovascularization of the infarct, leading to improvements in LV functional performance after the occurrence of ischemic heart disease.

We add a cautionary note regarding the possible consequences of altering the fibrosis process after myocardial infarctions. The evolution of composition in myocardial tissue from infarction to scar formation passes through a phase of cellular necrosis, which creates a soft fragile region of myocardium susceptible to rupture should the aortic and consequently ventricular systolic pressure become markedly elevated from any cause [7]. Similarly, approaches to reduce or reverse fibrosis and collagen formation in scar could present similar risk, however no such adverse events were noted in the current study.

## Conclusion

This study demonstrated that miR-29b overexpression is an emerging supplement to iPSC-based cell therapy for ischemic heart disease. MiR-29b overexpression reduces the barrier (collagen deposition) to iPSC engraftment from epicardial apposed myocardial tissue progenitor cell patches. In combination with the Tri-P, overexpression of miR-29b was accompanied by enhanced angiomyogenesis in the underlying infarct region and functional restoration of the LV after MI.
